# Snowflake Data Warehouse for Large-Scale and Diverse Biological Data Management and Analysis

**DOI:** 10.3390/genes16010034

**Published:** 2024-12-28

**Authors:** Tatsuya Koreeda, Hiroshi Honda, Jun-ichi Onami

**Affiliations:** 1 CLINIC FOR Group, Nagisa Terrace 4F, 3-1-32 Shibaura, Minato-ku, Tokyo 108-0023, Japan; 2 Kao Corporation, Bunka, Sumida-ku, Tokyo 131-8501, Japan; 3 RIKEN BioResource Research Center, 3-1-1 Koyadai, Tsukuba 305-0074, Japan

**Keywords:** big data, biodata, bioinformatics, data science, data warehouse, Snowflake

## Abstract

With the increasing speed of genomic, transcriptomic, and metagenomic data generation driven by the advancement and widespread adoption of next-generation sequencing technologies, the management and analysis of large-scale, diverse data in the fields of life science and biotechnology have become critical challenges. In this paper, we thoroughly discuss the use of cloud data warehouses to address these challenges. Specifically, we propose a data management and analysis framework using Snowflake, a SaaS-based data platform. We further demonstrate its convenience and effectiveness through concrete examples, such as disease variant analysis and in silico drug discovery. By introducing Snowflake, researchers can efficiently manage and analyze a wide array of biological data, enabling the discovery of new biological insights through integrated analysis. Through these specific methodologies and application examples, we aim to accelerate research progress in the field of bioinformatics.

## 1. Introduction

**Figure 1:**
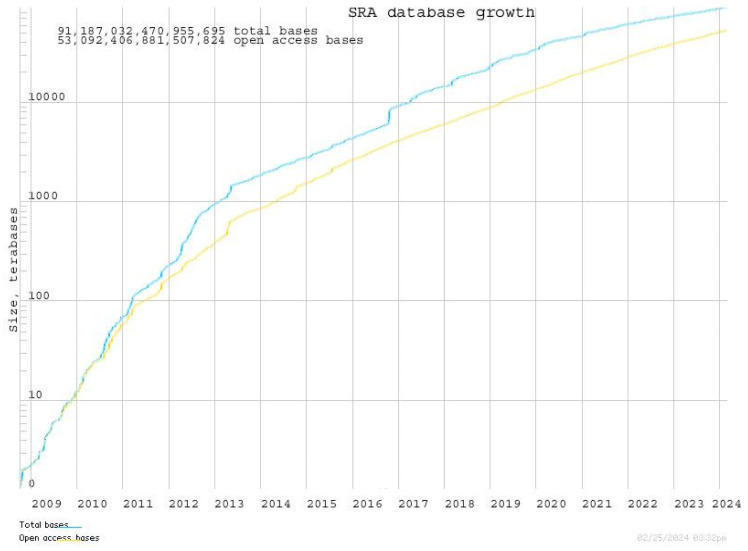
Annual increase in the capacity of Sequence Read Archive (SRA) data released by the National Center for Biotechnology Information (NCBI). The graph illustrates a dramatic growth trend from 2009 to 2024, increasing approximately 620,000 times in total base pairs. This growth reflects the advancements in sequencing technologies and the increasing volume of genomic data available for research. Such exponential growth underscores both the opportunities and challenges in managing and analyzing large-scale biological datasets. The figure is cited from [5].

Advances in sequencing technology have led to a rapid increase in the pace of genomic data production, and as a result, the cost of data production is decreasing dramatically [1,2]. For example, the capacity of the Sequence Read Archive (SRA) data published by the National Center for Biotechnology Information (NCBI) was 47.04 GB in May 2007, but by February 2024, it had grown to 27.93 PB, an increase of approximately 620,000 times (Figure 1). Thanks to technological innovations by Illumina, Inc., the cost of sequencing a human genome fell below $1000 in 2014 and is now reported to have dropped to $600 [3]. While this rapid increase in data and the decrease in costs have made it possible to apply sequencing technologies to a wider range of research and clinical applications, they have also introduced new challenges for modern researchers in the management and analysis of biological data [4].

**Figure 2:**
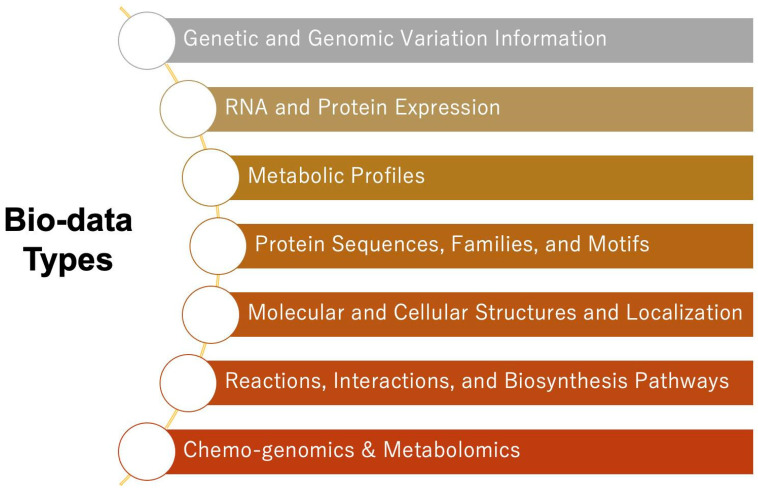
This figure lists the diverse types of biological data, which include gene and genome mutation information, RNA and protein expression, metabolic distributions, protein sequences, families and motifs, molecular and cellular structures and localization, reactions, interactions, and biosynthetic pathways. Additionally, emerging fields such as chemogenomics and metabolomics are included, showcasing the vast and interdisciplinary nature of biological datasets. This diversity underscores the necessity for integrated data management and analysis platforms to address the challenges posed by large-scale and heterogeneous datasets.

In addition to genomes and genes, the types of biological data are diverse. For example, the variety of data have significantly expanded to include information on gene and genome mutations, RNA and protein expression, metabolic distributions, protein sequences, families and motifs, molecular and cellular structures and localization, reactions, interactions, biosynthetic pathways, and even fields such as chemogenomics and metabolomics [6,7,8] (Figure 2). Moreover, not only structured data like aligned tables and charts exist, but also unstructured data, such as medical diagnostic information stored in PDFs or pathology-stained images [9,10]. Furthermore, research laboratories in companies possess proprietary seed information (information on safety test results, pharmacology, pharmacokinetics, efficacy, and formulation of synthetic compounds, etc.), and in recent years, virtual data obtained through the use of modeling and simulation has been utilized. Modern researchers are thus required to properly manage and analyze massive and diverse biological data in an integrated manner to gain new insights. As a result, the increasing storage management and operational costs associated with analysis have become a challenge [11]. Advances in bioinformatics are crucial for the development of biological data management and analysis, and the improvement of efficient data management and analysis techniques is expected to promote growth in the field of bioinformatics and the dissemination of technology to researchers. Recently, cloud platforms have been used for managing and analyzing biological data [12,13]. Cloud technologies are widely utilized for analyses in bioinformatics due to their scalability, flexibility, and cost-effectiveness [14,15]. This paper focuses on cloud technologies, particularly data warehouse (DWH), as a platform for efficiently managing and analyzing large-scale and diverse biological data. Our aim is to establish a cost-effective and efficient analytical infrastructure for biological data, facilitating breakthroughs in bioinformatics research.

## 2. Comparison of On-Premises, Supercomputers, and Cloud Computing

The primary storage and computing resources used in academic research include personal laptops, lab-owned desktop PCs and servers (on-premises environments), and supercomputers (Table 1). One advantage of on-premises environments is the ability to freely customize both hardware and software. However, initial investments are required for hardware, software, and environment setup. Additionally, the cost of acquiring high-spec CPUs, GPUs, and memory can be significant, and the maintenance and upgrading of these systems can be challenging, requiring advanced IT skills [16]. On the other hand, supercomputers, with their high computational power, are ideal for large-scale, complex simulations and data analyses. They allow for the optimization of system performance tailored to specific computational tasks. However, supercomputers often require reservations for shared use, and access time may be limited [17]. Specifically, it is challenging to continuously store large amounts of biological data in such systems, and important or confidential company data cannot always be handled due to security concerns. Additionally, using a supercomputer involves various administrative tasks, such as submitting application forms, undergoing reviews, providing performance reports, attending reporting meetings, and managing procedures during organizational changes. Given these limitations, cloud technology offers an effective solution (Table 1).

Cloud computing refers to servers, databases, and software accessed via the internet. This allows users to create virtual machines on resources managed by vendors, without needing to own physical hardware. Consequently, users can flexibly scale computing resources up or down based on their needs, enabling them to quickly build high-performance computing (HPC) clusters for large-scale simulations and data analyses, all while keeping costs lower than on-premises setups. Cloud platforms typically use a pay-as-you-go model, where users are charged only for the resources they use. In recent years, universities have made payments easier by accommodating invoicing through resellers, lowering the barriers to adoption. Additionally, cloud providers bear the costs of hardware maintenance and upgrades.

For example, Amazon Web Services (AWS) is responsible for operating, managing, and controlling a wide range of components, from the host operating system and virtualization layer to the physical security of the data centers where services operate [18]. However, the extent of user responsibility varies by service. For instance, users of Amazon EC2 are responsible for tasks such as updating the guest OS and performing other administrative duties, whereas services like Amazon S3 involve minimal user management, with AWS handling most of the responsibilities. Furthermore, cloud providers offer robust security and compliance features, including data encryption, access control, and audit logs, ensuring the safe management of sensitive biological data such as genomic data and patient information. Cloud providers support compliance with international and regional regulations like GDPR (General Data Protection Regulation) and HIPAA (Health Insurance Portability and Accountability Act), making it easier to ensure legal compliance. In such an environment, data sharing and collaboration are also simplified, enabling easy sharing of data and analysis results. This allows multiple researchers and teams to access the data simultaneously, improving research efficiency.

While cloud technology offers many advantages, there are also some drawbacks. First, regarding operational costs, the pay-as-you-go model is suitable for short-term use but storing large-scale data over long term can sometimes become more expensive than on-premises solutions. Additionally, there is the issue of vendor lock-in; relying on a single provider can lead to extra costs and complications when migrating to another cloud service. Moreover, although the risk of service discontinuation is low, it is advisable to implement data backups and adopt a multi-cloud strategy as a precaution.

## 3. About DWH Products

**Figure 3:**
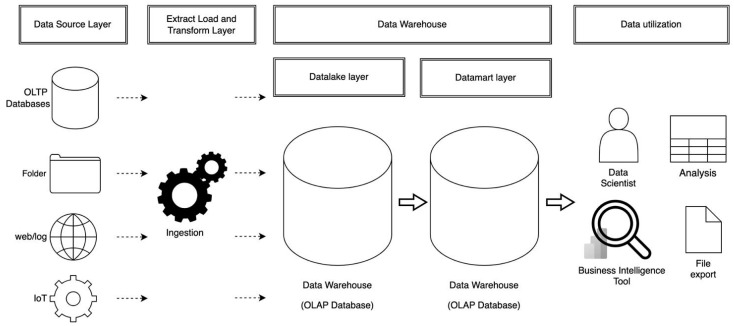
Data management system centered around a data warehouse. An example of a data management system centered around a cloud DWH product. Starting from the data source layer, which includes OLTP databases, folders, web/logs, and IoT data, the ELT layer is responsible for data extraction and transformation. Within the DWH, data are stored in the data lake layer, followed by the data mart layer, where it is processed and organized into a more accessible format. Finally, data scientists and BI tool users can utilize the data for analysis and insights.

A DWH is a system designed for centralized management of large amounts of data and is commonly used by companies to integrate disparate data sources, supporting business intelligence and advanced analytics [19,20,21]. By using DWH, organizations can centrally manage processes from data ingestion, transformation, and storage to analysis, ensuring data consistency and quality while enabling efficient data management (Figure 3). The data modeled in the DWH is managed as a data mart layer, which is analyzed and visualized by data utilization layers, such as data science approaches or business intelligence (BI) tools, including graph visualization tools [22]. Additionally, cloud-hosted DWHs fully leverage the benefits of the cloud, including scalability, flexibility, cost efficiency, data sharing and collaboration, as well as security and compliance [23]. Nowadays, in addition to data management, DWHs offer enhanced capabilities for big data analytics, AI, and machine learning, allowing organizations to carry out end-to-end processes, from data management and modeling to visualization and analysis [24].

One of the key features of DWH products is their support for On-Line Analytical Processing (OLAP). OLAP is a columnar database technology that enables the rapid aggregation of large datasets and excels in multidimensional data modeling, allowing for analysis across various segments [25]. OLAP is often contrasted with On-Line Transaction Processing (OLTP). OLTP is a database technology designed to automate transaction processing, ensuring atomicity, consistency, isolation, and durability (ACID properties). OLTP typically handles current, detailed data and performs fine-grained I/O operations on a small number of records. The functional and performance requirements of DWHs differ significantly from those of traditional operational databases. OLTP is designed to ensure that transactions adhere to ACID properties, automating data processing tasks such as software requests. These transactions require detailed and up-to-date data, usually accessed via primary keys, and perform fine-grained I/O operations on a few records. OLTP focuses on ensuring consistency and recoverability, with the goal of maximizing transaction throughput. In contrast, OLAP is designed for decision support. OLAP typically manages data that have been aggregated over long periods from multiple operational databases, resulting in datasets that are orders of magnitude larger than those used in OLTP. By using SQL and other Data Manipulation Languages, OLAP can scan, join, and aggregate millions of records, where query response time is a more critical metric than transaction throughput.

## 4. Advantages of Biological Data Management in the Snowflake

Snowflake, a cloud-hosted DWH product, offers several advantages for biological data management. Compared to other DWH products such as Google BigQuery [26], and Amazon Redshift, Snowflake stands out in several areas (Table 2). Notably, it has a high level of compatibility with the three major cloud platforms (AWS, GCP, and Azure) [27], allowing flexible data management while avoiding the risk of vendor lock-in. This flexibility is crucial for researchers managing data across multiple cloud environments. In addition, Snowflake’s near-zero maintenance design [28] enables automatic infrastructure optimization, significantly reducing the operational burden and allowing researchers to focus on their core analytical tasks. Furthermore, Snowflake simplifies data ingestion across different cloud environments, such as S3 and GCS, which lowers collaboration costs in biological data management across clouds. This results in greater flexibility and efficiency in multi-omics analysis compared to BigQuery and Redshift. Snowflake’s Secure Data Sharing feature allows users to securely share data with other users or organizations without the need for physical copies [29]. Moreover, its zero-copy clone functionality [30], enables the creation of data clones without physically duplicating the data, allowing researchers to perform analysis and validation through references to the original dataset. However, these vendor-specific features, such as zero-copy cloning and Secure Data Sharing, may introduce challenges in interoperability with other platforms or when migrating datasets to alternative systems. Researchers must carefully plan for long-term data portability when incorporating such features into their workflows. This not only improves the efficiency of biological data analysis but also reduces storage costs. In contrast, BigQuery and Redshift often require physical copies for data sharing and replication, potentially increasing security risks and management costs. While specific cost comparisons with competitors such as Google BigQuery and Amazon Redshift are not included in this discussion, Snowflake’s cost-effectiveness stems from its ability to optimize overall storage and computation costs. Features like zero-copy cloning and cross-cloud data ingestion enable efficient handling of large-scale biological datasets, particularly in collaborative and multi-cloud scenarios. Additionally, for researchers unfamiliar with cloud-based platforms, there may be a learning curve associated with adopting Snowflake’s advanced capabilities. Snowflake mitigates this challenge through its robust training programs and active user community, which support skill development and smooth adoption. Snowflake’s features are particularly valuable in bioinformatics and healthcare fields where privacy and security are critical. These capabilities provide enhanced flexibility and efficiency in managing biological data while keeping storage costs low.

While Snowflake offers outstanding features for biological data management, BigQuery and Redshift also have their own advantages. BigQuery’s strength lies in its native support for genome annotation datasets, such as TCGA [31], ClinVar [32], and COSMIC [33]. Additionally, BigQuery seamlessly integrates with a variety of AI and machine learning tools, including Google’s AI platform (Vertex AI) and tools like AlphaFold2, which excel in genome analysis and protein folding prediction. On the other hand, Redshift offers easy integration with genomic workflows within the AWS ecosystem, enabling efficient genomic data analysis and pipeline management using tools like AWS Batch and SageMaker.

## 5. Biological Data Management and Analysis Framework in the Snowflake

**Figure 4:**
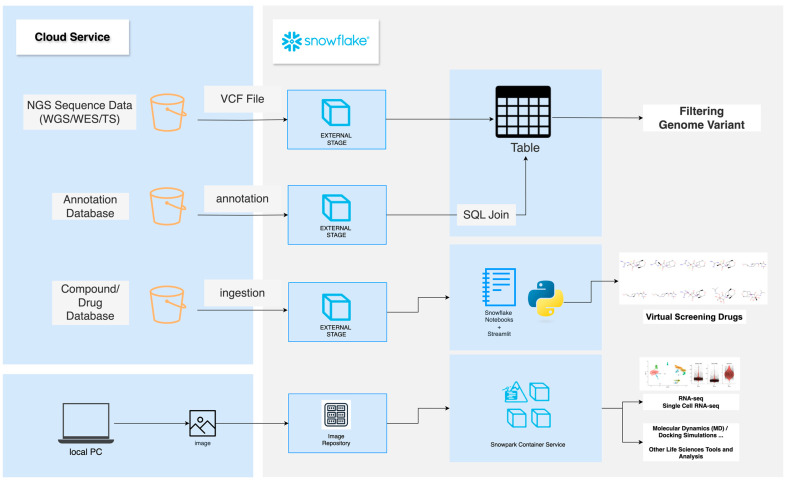
Biological Data Management and Analysis Using Snowflake.

In recent years, bio-researchers have increasingly been required to conduct integrative research by combining multiple biological sources for analysis. Multi-omics analysis is a method that integrates and analyzes various omics data, such as genomics, proteomics, transcriptomics, and metabolomics. Multi-omics analysis provides comprehensive biological insights that cannot be obtained from a single omics dataset alone. Using a DWH allows researchers to centrally manage these different types of biological data, ensuring security while enabling easy access for analysis, visualization, and sharing. We have been using Snowflake as a DWH to establish a multi-omics analysis environment [34]. Here, we propose a biological data management method and a specific analysis framework using Snowflake (Figure 4). The pricing structure of Snowflake is beyond the scope of this discussion; readers are encouraged to refer to the official service documentation for further details [35].

An example of a biological data management and analysis platform using Snowflake as a DWH. From top to bottom, it illustrates disease genome variant filtering analysis, in silico drug discovery analysis such as virtual screening, and transcriptome analysis, as well as other high-performance computing analyses hosted using OCI images. Cloud storage is integrated via Snowflake’s external stage feature, enabling seamless integration with external cloud storage services. The vendor logos for Snowflake are used in accordance with each company’s brand guidelines.

### 5.1. Disease Variant Analysis

Variant Call Files (VCFs) typically range from 2 to 10 GB per file, and the increase in storage requirements when managing multiple samples presents a significant challenge. Snowflake supports the management of VCFs as unstructured data with built-in storage compression capabilities [36]. Since the number of variants in a human genome often exceeds several million, leveraging OLAP’s performance is ideal for efficient management. Disease variant analysis is the process of identifying patient-specific genomic mutations using next-generation sequencing (NGS) data to pinpoint disease-related variants. One example of an analysis workflow involves using VCF files from the 1000 Genomes Project, which have been processed by DRAGEN (Dynamic Read Analysis for GENomics) and are available through the Registry of Open Data on AWS [37]. As of July 2024, the Registry of Open Data on AWS hosts 544 datasets, including 60 genome- and gene-related datasets, publicly available on AWS S3 [38]. Snowflake’s external stage feature allows S3 or other external storage to be registered as part of Snowflake’s external storage system [39]. Afterward, VCF files can be ingested into tables using pre-defined User-Defined Functions (UDFs). Once ingested, the variant information can be filtered using SQL, the database query language. The Registry of Open Data on AWS also provides annotation databases such as gnomAD [40,41] and panels data [42], which describe the geographic origins, gender, and familial relationships of samples. These datasets can be SQL-joined with the variant information stored in tables to perform more detailed variant filtering.

### 5.2. In Silico Drug Discovery (Virtual Screening)

In silico drug discovery typically involves the process of searching for promising similar compounds based on information published in patents and research papers. Snowflake allows for the ingestion of compound and drug data stored on cloud storage, or files downloaded from databases such as ChEMBL [43], PubChem [44], or ZINC15 [45]. These compounds or drug data can be stored in Snowflake’s internal stage and analyzed using developer toolkits. Snowflake offers a Jupyter Notebooks-like GUI analysis feature called Snowflake Notebook [46] where informatics libraries like RDKit and Biopython, mirrored from Anaconda packages, are supported by default. Additionally, screened compounds and drug data can be visualized using Streamlit in Snowflake, enabling structural information visualization [47]. From data ingestion to analysis and visualization, researchers can build a workflow for identifying potential drug candidates without exporting data outside of Snowflake. To illustrate the practical applications of Snowflake in in silico drug discovery, consider the following workflow: Using the ZINC database, researchers can screen for compounds similar to neuraminidase inhibitors like laninamivir. The workflow involves loading compound data into Snowflake’s internal stage, calculating molecular similarities using RDKit within Snowflake Notebooks, and visualizing the results with Streamlit. For example, compounds with a Tanimoto similarity score above a certain threshold can be identified and ranked. This integrated approach allows researchers to efficiently analyze, filter, and visualize compound data without transferring data outside Snowflake. Such workflows demonstrate Snowflake’s potential in streamlining the early stages of drug discovery.

### 5.3. High-Performance Computing Resource Analysis Using OCI Image

Snowflake supports Snowpark Container Service (SPCS), a fully managed container product that simplifies the deployment, management, and scaling of containerized applications within the Snowflake ecosystem [48]. By leveraging SPCS, many life sciences tools can be used on Snowflake using Open Container Initiative (OCI) images, including Docker-based container runtime technologies. For example, to perform single-cell RNA sequencing (RNA-seq) analysis, an RStudio Server image can be pushed from a local PC to an image repository. By hosting this image in SPCS, an R environment with an endpoint can be set up, enabling single-cell RNA-seq analysis using the Seurat package. SPCS supports up to 1024 GiB of CPU memory and also supports NVIDIA GPUs, making it possible to perform end-to-end analysis for high-performance computing tasks such as molecular dynamics (MD) and docking simulations.

By utilizing this framework, researchers can fully leverage the advantages of a DWH centered on Snowflake, enabling effective management and analysis of biological data. The aforementioned analyses, ranging from genome analysis to cheminformatics and transcriptome analysis, are all managed within Snowflake, making integrated analysis straightforward. Centralized data management combined with the integration of diverse analytical tools expands the possibilities for advancements in bioinformatics.

## 6. Use Cases of DWHs in Life Sciences Companies

In the previous section, we discussed the general use of DWHs in bioresearch. However, DWHs are also increasingly being used by private companies in the life sciences sector, such as pharmaceutical companies and medical device manufacturers, beyond internal data management. This section briefly introduces notable use cases of DWHs in life sciences companies, including the application of Data Clean Rooms and LLMs for processing life sciences data.

### 6.1. Data Clean Room

A Data Clean Room is a secure environment that allows multiple organizations to share data while ensuring privacy and compliance, often using differential privacy techniques [49,50]. In the life sciences field, sensitive data such as patient records and clinical trial data are frequently shared between research institutions and pharmaceutical companies. By using a Data Clean Room, it is possible to anonymize data or share only the necessary portions, allowing organizations to collaborate on research and analysis while safeguarding data privacy.

### 6.2. Handling Life Sciences Data with LLMs

Large Language Model (LLM) is a machine learning model trained on vast amounts of text data, with the ability to understand and generate natural language [51]. It excels at processing and learning from large volumes of unstructured data. In the life sciences field, a significant amount of unstructured data exists, such as research papers, patents, and electronic health records. By leveraging LLMs, these data can be efficiently analyzed, leading to the extraction of new insights [52]. For instance, LLMs can be used to analyze clinical trial data to identify effective treatments or to detect risk factors for specific diseases from patient data [53].

### 6.3. Snowflake as a Data Connection Hub and Its Scalability

Snowflake provides robust connectivity among ecosystems. It links various analytical workflows seamlessly or integratively. This saves the costs of controlling research data connection and tool construction across remote networks and on-premises environments through combinations of API schemas and various query values. Furthermore, extensive plugins are available in the Snowflake Marketplace [54] such as supporting tools for expanded SQL code and database connectors. A plugin for SPARQL graph data queries, frequently adopted in life science database research, can also be requested via the data world plug-in [55]. Research data in the life sciences field is comparatively diverse, and a lot of databases are freely open and highly interconnected [56]. It indicates that Snowflake’s connectivity for research analysis is effective.

## 7. Conclusions

Cloud-based bioinformatics is a powerful tool for managing and analyzing large-scale, diverse biological data. By using cloud-based DWHs like Snowflake, researchers can enhance the scalability, flexibility, cost-efficiency, and security of data management, enabling advanced analysis of large and complex biological datasets. I hope that the examples of analyses and the multi-omics environment setup introduced in this paper have demonstrated the potential of cloud-powered bioinformatics. With continued advancements in cloud technology, further discoveries and progress in the field of bioinformatics are expected.

## Figures and Tables

**Table 1:** Comparison of On-Premises, Supercomputers, and Cloud Computing.

Criteria	On-Premises	Supercomputers	Cloud Computing
Customization	Free hardware/software customization	Performance optimization for specific tasks	Dynamic resource scaling
Cost	High maintenance/operation costs	Utilization limits and administrative overhead	Pay-as-you-go model but potential long-term cost increases
Performance	Dependent on the hardware investment	High performance for large-scale computations	High performance with scalable virtual clusters
Flexibility	Limited by physical resources	Requires shared use and reservations	Flexibility in scaling resources
Security	Managed internally	Potential concerns for handling sensitive data	Robust security with compliance (GDPR, HIPAA)
Data Sharing	Not typically optimized for sharing	Challenging for long-term data storage	Easy sharing and collaboration on a global scale
Risks	High maintenance and operational burden	Limited usage windows, complex management	Vendor lock-in, risk of service termination

**Table 2:** Comparison of DWH for Biological Data Management Platforms.

Criteria	Snowflake	Google BigQuery	Amazon Redshift
Cloud Compatibility	High compatibility with AWS, GCP, and Azure. Avoids vendor lock-in, enabling flexible data management.	Runs on Google Cloud Platform, dependent on GCP.	Primarily dependent on AWS, making multi-cloud operations challenging.
Maintenance Burden	Near-zero maintenance design with automated infrastructure optimization.	Serverless with minimal maintenance, but less infrastructure flexibility.	Regular maintenance required, with more manual configuration and scaling needed.
Data Ingestion Flexibility	Easy data ingestion between different cloud environments (e.g., S3, GCS).	Optimized for GCS ingestion but limited integration with other clouds.	Best optimized for S3 data ingestion, but integration with other clouds is difficult.
Data Sharing	Secure Data Sharing feature allows secure sharing of data with other users or organizations without physical copies.	Often requires physical data copies, leading to higher sharing costs and time.	Data sharing requires physical copies, which can increase security risks and management costs.
Data Cloning	Zero-copy cloning allows for the creation of clones without actual data replication.	Limited cloning capabilities, often requiring physical data copies.	Physical data copies are required for data cloning, leading to increased management overhead.
Biological Data Management Flexibility	Highly flexible in cross-cloud collaboration and biological data management.	Dependent on GCP, with limited flexibility but strong scalability.	AWS-dependent, with limitations in flexibility for cross-cloud biological data management.

## Data Availability

This review article partially includes translated content from a JSBi Bioinformatics Review manuscript https://doi.org/10.11234/jsbibr.2024.primer2 (accessed on 25 December 2024). In accordance with the Creative Commons Attribution-NonCommercial-ShareAlike 4.0 International (CC BY-NC-SA 4.0) license, translation into other languages and reuse of figures and tables are permitted. This manuscript is submitted in compliance with the journal’s redistribution guidelines.
